# The Modulation of Stimulus Familiarity on the Repetition Effect in Duration Judgment

**DOI:** 10.3389/fpsyg.2020.01181

**Published:** 2020-06-12

**Authors:** Lina Jia, Can Deng, Lili Wang, Xuelian Zang, Xiaocheng Wang

**Affiliations:** ^1^Department of Education, School of Humanities, Jiangnan University, Wuxi, China; ^2^Department of Psychology, Huaiyin Normal University, Huai’an, China; ^3^School of Education, Institutes of Psychological Sciences, Hangzhou Normal University, Hangzhou, China

**Keywords:** duration judgment, repetition compression, constituent repetition, Chinese, predictive coding

## Abstract

The repetition of a stimulus often produces a shorter subjective duration than does the presentation of a novel item. To test whether familiarity mediates the repetition compression effect, the present study compared the influence of repeated words and pseudowords on apparent duration, using a duration discrimination task. We found a similar magnitude of temporal compression for the repeated-word and repeated-pseudoword conditions. When introducing a further experiment with two new conditions in which the standard–comparison pair shared a character at the first or second constituent position, we observed a shorter subjective duration for whole word (or whole pseudoword) repetition compared with the remaining conditions (i.e., first-character repetition, second-character repetition, and novel baseline). However, temporal compression for the first- and second-character repetitions was observed only for pseudowords but not for words. Our findings indicate that familiarity modulates the perception of duration in constituent character repetition. The results are discussed on the basis of the predictive coding theory.

## Introduction

Prior experience with a given stimulus ultimately affects how it is perceived, usually resulting in faster reaction times and/or better accuracy to judge the repeated items (Tenpenny, 1995). This effect is observed with various visual and auditory stimuli ranging from simple to complex forms. Importantly, and central to this study is that prior experience has also been shown to influence a range of temporal judgment tasks, where the presentation of a stimulus is usually perceived as shorter in duration when it is a repeat of the preceding item (Matthews, 2011; Birngruber et al., 2015; Matthews and Gheorghiu, 2016). Despite the fact that the foundation for the repetition effect on time duration still remains a subject of debate, three main accounts have been put forward. These can be subdivided into two main theoretical approaches (Matthews et al., 2014; Matthews and Gheorghiu, 2016; Ulrich and Bausenhart, 2019). The first theoretical approach includes the attentional and arousal accounts, both derived from the pacemaker-accumulator framework, which assumes an inherent timing process, referred to as the classical internal clock model (Treisman, 1963; Gibbon, 1991; Wearden, 1992; Zakay and Block, 1997). In this model, a switch connects the pacemaker and the accumulator by switching on and off to count the pulses generated by the pacemaker. Under this framework, novel stimuli are more arousing and/or capture more attention, thus accelerating the pacemaker, which generates more temporal pulses. As a result, the total number of pulses (and their respective intervals) counted by the accumulator leads to an overestimation of perceived duration (Tse et al., 2004; Ulrich et al., 2006; Ciria et al., 2019). To illustrate, using the oddball paradigm, Tse et al. (2004) found that an oddball stimulus (e.g., expanding disk) embedded in a train of repeated stimuli (e.g., black stationary disk) was perceived as longer than the repeated ones. The authors concluded that the repetition effect depended on the orientation of attention in response to the relative saliency of the stimuli.

The second theoretical approach is based on neurophysiological findings where reduced neural activity was observed in response to repeated stimuli, a phenomenon referred to as repetition suppression (Desimone, 1996; Henson and Rugg, 2003; Grill-Spector et al., 2006; Eagleman and Pariyadath, 2009). This effect leads to a shortened apparent duration of repeated stimuli compared to novel ones (Birngruber et al., 2015; Cai et al., 2015). Accordingly, the repetition suppression could be due to a low-sensory adaptation or a more efficient representation (Desimone, 1996; De Baene and Vogels, 2010). The latter relates to the framework of predictive coding (Matthews et al., 2014) wherein prior exposure produces a prediction that the same stimulus will recur. In order to perform efficient coding, the brain does the minimum necessary to process repeated stimuli and only thoroughly processes the stimulus deviant from the implicit expectation, a process referred to as surprising prediction errors (Rao and Ballard, 1999; Friston, 2005; Eagleman and Pariyadath, 2009). In other words, neural responses are suppressed for the predicted signals (repeated stimuli) and activated or even enhanced for the surprising stimuli (oddball/novel stimuli). The view of predictive coding was supported by a large number of studies (Matthews, 2011; Schindel et al., 2011; Pariyadath and Eagleman, 2012; Birngruber et al., 2015; Jia and Shi, 2017; Saurels et al., 2019). In this framework, the size of the repetition effect should be dependent on the similarity between the predicted and actual signals. For instance, the number of changes (e.g., varying the angles of lines) between the oddball and standard stimuli has been shown to modulate the magnitude of the oddball effect (Schindel et al., 2011; Pariyadath and Eagleman, 2012). However, more recent studies found that the repetition duration compression was reduced or even reversed when repeats were frequent compared with when repeats were rare across blocks, indicating an interaction between low-sensory adaptation and high-level expectation (Matthews, 2015; Skylark and Gheorghiu, 2017).

An additional branch of research has examined the influence of familiar (e.g., word) or unfamiliar stimuli (e.g., pseudowords) on the repetition effect over duration perception. It has been shown that both categories are prone to duration compression in relation to novel items (Matthews, 2011, 2015; Birngruber et al., 2015; Cai et al., 2015; Jia and Shi, 2017). As an example, in a duration discrimination task, in which the participants had to respond whether the presentation time of the comparison stimulus was shorter or longer than that of the baseline, Birngruber et al. (2015) found that the presentation of repeated unfamiliar pseudowords was judged as shorter in duration than that of a novel baseline stimulus. Along with a similar method, namely, two-interval paradigm, Jia and Shi (2017) showed that participants tended to underestimate the apparent duration of the repetition of familiar stimuli (i.e., Chinese character).

Although the aforementioned studies demonstrated that the repetition of familiar or unfamiliar stimuli produces duration compression relative to novel stimuli (Matthews, 2011; Birngruber et al., 2015; Cai et al., 2015; Jia and Shi, 2017), it is vital to highlight that there has been little evidence of how the familiarity (i.e., words vs. pseudowords in the same study) influences the size of the repetition duration effect. Specifically, the question is to what extent familiarity interacts with the repetition duration effect. To date, a few studies employing lexical decision and identification tasks have shown that the effect caused by the repetition of familiar stimuli was more pronounced than that of unfamiliar stimuli (Henson et al., 2000; Fiebach et al., 2005; Orfanidou et al., 2006). Moreover, neuroimaging studies revealed that as neural responses to repeated-familiar stimuli (e.g., famous faces and words) decreased, the neural responses to repeated-unfamiliar stimuli (e.g., non-famous faces and pseudowords) increased (Henson et al., 2000; Fiebach et al., 2005). Nevertheless, it is worth noting that the tasks mentioned above involved access to semantic representations in the high-level hierarchy. These tasks differ from duration judgment tasks in the sense that the latter do not require semantic-related judgment but rather judge the speed of time passage of a stimulus. For this reason, it is essential to use duration judgment tasks to investigate whether stimulus familiarity mediates the repetition duration effect.

A further point fundamental to the current study concerns the interaction of stimulus familiarity with the repetition duration effect using constituent vs. whole stimulus representation. This is important because the extant line of investigation has mainly focused on the effects of whole-stimulus repetition (e.g., picture, simple symbol, and character). It is important to highlight that these studies demonstrated the repetition duration compression (Matthews and Gheorghiu, 2016) and explained the phenomenon with bases on the predictive coding account (Schindel et al., 2011; Pariyadath and Eagleman, 2012; Jia and Shi, 2017; Saurels et al., 2019). Under this account, the magnitudes of the repetition effect reflect the discrepancy between the predicted and actual items in terms of their physical characteristics. On this note, it is theoretically critical to compare the effects of whole repetition and constituent repetition in duration perception. In particular, the investigation of constituent repetition can bring to the fore how it interacts with stimulus familiarity. What is more, until now, most empirical evidence for the constituent repetition effect comes from studies using various non-temporal tasks (Zhou et al., 1999; Tsang et al., 2014; Wu et al., 2016), such as the study by Zhou et al. (1999), which found the repetition effect in an identification task even when the standard and comparison words shared only one constituent character (e.g., “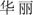-magnificent” vs. “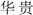-luxurious”). In the present study, however, we ask the question as to whether familiarity mediates the effect of constituent repetition in a duration judgment task.

To answer the questions aforementioned, the present study adopted Chinese two-character words and pseudowords. To examine the familiarity-by-repetition interaction in duration judgment, Experiment 1 compared the repetition effect of Chinese words with that of pseudowords in a duration discrimination task. With our experimental design, if repeated pseudowords produce duration compression similar to that of repeated words, then the magnitude of the repetition compression in duration is not mediated by stimulus familiarity. On the other hand, should duration compression be stronger for repeated words than that of repeated pseudowords, we can argue that familiarity does interact with the effect. In Experiment 2, we explore whether the apparent duration is compressed when the standard–comparison pair shares one constituent character and whether the constituent repetition effect is affected by familiarity. More specifically, in Experiment 2a, we administer four experimental conditions: whole-word repetition, first-character repetition, second-character repetition, and novel baseline. In Experiment 2b, we adopt the same design used in Experiment 2a and test the constituent repetition effect for pseudowords containing two unrelated characters. Note that we designed the first- and second-character repetition as two separate conditions to avoid inconsistent results regarding the interaction between constituent position and repetition effect as reported in previous studies (Zhou et al., 1999; Wu et al., 2016).

## Experiment 1

### Methods

#### Participants

Twenty students from Jiangnan University volunteered to take part in the experiment (six females; mean age 23.4 years). All participants were native speakers of Chinese, with normal or corrected-to-normal vision. All participants gave written informed consent before the experiment.

#### Stimuli and Apparatus

One hundred ten Chinese bimorphemic words and 110 pseudowords were selected to serve as the experimental stimuli. For words (e.g., 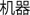 /ji 1 qi 4/, meaning “machine”), the average frequency was 44 words/million, and the mean of strokes was 16. Pseudowords (mean of strokes: 17) were constructed by combining the existing characters to generate non-existing and non-interpretable bimorphemic stimuli (e.g., 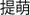/ti 2 meng 2/, no meaning). Words/pseudowords were equally divided into two groups (word, Group A_w_ and B_w_; pseudoword, Group A_p_ and B_p_).

Words in Groups A_w_ and B_w_ were matched for word frequency and the number of strokes (both *p*s > 0.1). Likewise, the number of strokes between the two groups of pseudowords was also matched (*p* > 0.1). The words from Group A_w_ served as the standard stimulus, which could be followed by either an identical word or a different comparison word from Group B_w_. The method used for pseudoword presentations was similar to the presentation of words. A 2 (repetition: repeated vs. novel) × 2 (word type: word vs. pseudoword) repeated-measures design was used in the experiment. The words/pseudowords (about 2.9° × 2.3° in size at the 57-cm viewing distance) were presented on a 24-in. LCD monitor with a refresh rate of 100 Hz. All visual stimuli were displayed in white on a black background. The left- and right-arrow keyboard keys were used as response keys. Each participant performed the experiment individually in a dimly illuminated room. The experiment was programmed with Matlab using the Psychophysics Toolbox (Brainard, 1997).

#### Procedure

**FIGURE 1:**
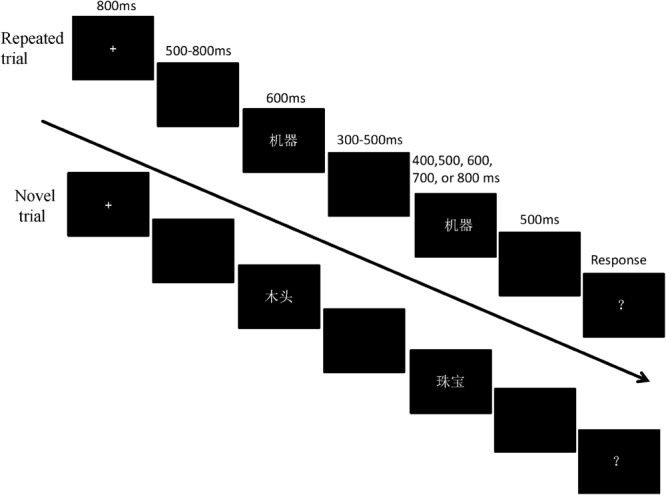
Schematic illustrations of stimuli presentation in Experiment 1. In the repeated trials, the standard and comparison words/pseudowords were identical (e.g., 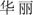/ji 1 qi 4/, meaning “machine”). In novel trials, the standard and comparison words/pseudowords were different (e.g., the standard: 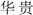/mu 4 tou 1/, meaning “wood”; the comparison: 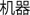/zhu 1 bao 3/, meaning “jewelry”).

The experiment adopted a classic duration comparison task. An illustration of the stimulus presentation is shown in Figure 1. Each trial started with a fixation cross, presented for 800 ms in the center of the screen, thereafter replaced with a blank interval of 500–800 ms. In the sequence, the standard stimulus was displayed for a fixed duration of 600 ms, followed by a 300–500 ms interstimulus interval (ISI). Next, the comparison stimulus was presented for one of the five possible durations – 400, 500, 600, 700, or 800 ms – which was followed by a 500 ms blank interval. Finally, the onset of a question mark (“?”) indicated that the participant should respond by pressing the left-arrow key if they perceived the duration of the comparison stimulus as being shorter than that of the standard stimulus, or the right-arrow key if they thought the comparison duration was longer. The intertrial interval (ITI) was set to 2,000 ms. All conditions were presented in random order, across 10 experimental blocks of 44 trials (22 times for each experimental condition). The words/pseudowords (each was used twice as the comparison stimulus) were randomly assigned to the comparison durations in each condition. The participants could take a break between blocks. Prior to the experimental session, each participant took part in a practice session of two blocks of 20 trials each. The test session lasted about 50 min.

### Results

We calculated the mean proportions of “longer” judgments (i.e., the comparison durations that were perceived as longer than the standard one) for each one of the five comparison durations, separately for each experimental condition and each participant. Thereafter, we fitted the psychometric curves to these proportions by using a logistic function. From the fitted curves, the point of subjective equality (PSE) and the just-noticeable difference (JND) were estimated. The PSE was the comparison duration at the 50% point of the curve as a measure of perceived duration (Treutwein and Strasburger, 1999; Birngruber et al., 2015; Jia and Shi, 2017). A lower PSE means longer perceived duration. The JND measure, as an index of discrimination sensitivity, was calculated by a half of the duration difference between the 25 and 75% points of the fitted response curves. A lower JND indicates better temporal discrimination. The unit of JND is represented in milliseconds.

**FIGURE 2:**
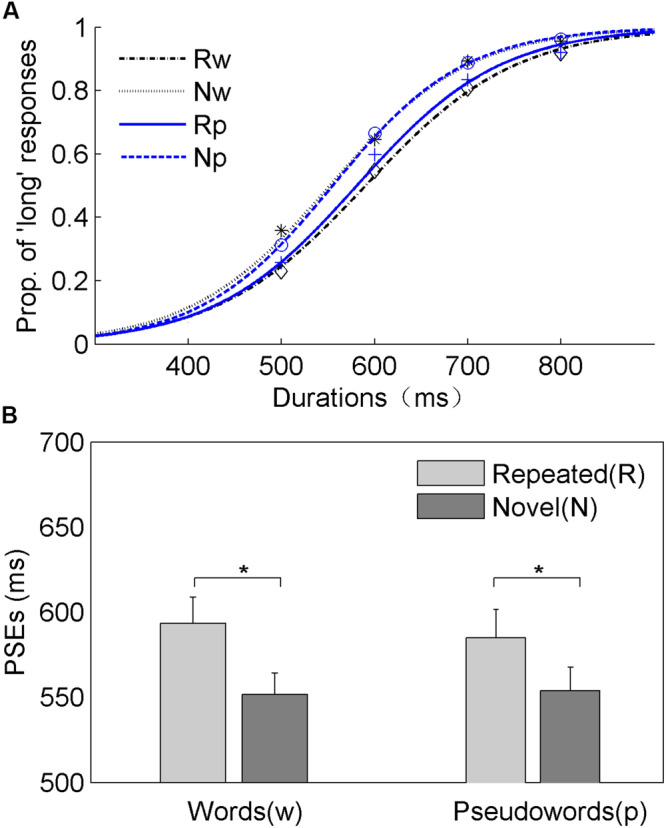
Results of Experiment 1. **(A)** Mean proportions of “long” responses against the comparison durations plotted with fitted psychometric curves representing the Rw, Nw, Rp, and Np conditions. **(B)** The mean points of subjective equality (PSEs) and respective standard errors are shown for the four conditions. *p < 0.05.

The mean PSEs (±SE) were 594 (±15), 552 (±12), 585 (±16), and 554 (±13) ms for repeated-word (“Rw”), novel-word (“Nw”), repeated-pseudoword (“Rp”), and novel-pseudoword (“Np”) conditions, respectively. Figure 2 shows the psychometric curves of the duration comparison task and the mean PSEs for all four experimental conditions. A repeated-measures ANOVA with the factors repetition (repeated vs. novel) and word type (word vs. pseudowords) was conducted on PSEs and JNDs. Regarding the analysis of the PSEs, the results revealed a significant main effect of repetition, *F*(1, 19) = 5.40, *p* < 0.05, η*_p_*^2^ = 0.22. The higher PSEs observed in the repeated condition (with respect to the novel condition) indicate duration compression. Neither the main effect of word type, *F*(1, 19) = 0.39, *p* = 0.54, η*_p_*^2^ = 0.02, nor their interaction, *F*(1, 19) = 2.62, *p* = 0.12, η*_p_*^2^ = 0.12, reached statistical significance. As for the analysis of JNDs, however, no significant effect was found [repetition, *F*(1, 19) = 0.87, *p* = 0.36, η*_p_*^2^ = 0.04; word type, *F*(1, 19) = 1.19, *p* = 0.29, η*_p_*^2^ = 0.06; interaction, *F*(1, 19) = 0.06, *p* = 0.81, η*_p_*^2^ = 0.003]. The findings revealed in Experiment 1 showed that the repetition of words and pseudowords produces comparable duration compression, thus indicating that familiarity did not interact with the repetition duration effect. To further investigate whether familiarity mediates duration perception when a pair of stimuli share a common constituent character, Experiment 2 compares whole repetition, first-character repetition, and second-character repetition for words (Experiment 2a) and pseudowords (Experiment 2b).

## Experiment 2

### Methods

#### Participants

Thirty-nine students from Jiangnan University volunteered to take part in the experiments (20 in Experiment 2a and 19 in Experiment 2b; 24 females; mean age: 21.6 years). All participants were native speakers of Chinese, with normal or corrected-to-normal vision. Written informed consent was obtained from each participant before the experiments.

#### Stimuli and Apparatus

The experimental design, stimulus presentation, and apparatus were identical to those used in Experiment 1, except for the addition of the “constituent repetition” conditions (see below). Fifty-five bimorphemic words (e.g., 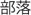/bu 4 luo 4/, meaning “tribe”) in Experiment 2a were selected to serve as standard stimuli, followed by one of four types of comparison stimulus: (i) identical word (e.g., 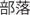), (ii) word with repetition of the constituent character at the first position (e.g., 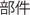/bu 4 jian 4/, meaning “component”), (iii) word with repetition of the constituent character at the second position (e.g., 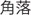/jiao 3 luo 4/, meaning “corner”), and (iv) novel word (e.g., 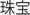/zhu 1 bao 3/, meaning “jewelry”). For the standard–comparison pairs sharing one character, the repeated characters had the same phonology and orthography. The comparison words were matched in frequency (mean 48 words/million) and in number of strokes (mean 15) across the four conditions (all *p*s > 0.1). The frequency and number of strokes between the shared characters in the first and second positions were also equal (both *p*s > 0.1). For easy reference, we labeled the four conditions in Experiment 2a as (i) word repetition (“Wr”), (ii) first-character repetition (“FCr”), (iii) second-character repetition (“SCr”), and (iv) novel baseline (“Nb”). The experimental conditions of Experiment 2b were identical to those of Experiment 2a, except that the stimuli used for duration judgment were pseudowords. Like Experiment 2a, the relevant conditions were matched in the number of strokes and character frequencies (all *p*s > 0.1).

#### Procedure

The experimental procedure adopted in Experiment 2 was identical to that used in Experiment 1, except for the experimental conditions, that is, four experimental conditions under word category (Experiment 2a) and another four experimental conditions under the pseudoword category (Experiment 2b).

### Results

**FIGURE 3:**
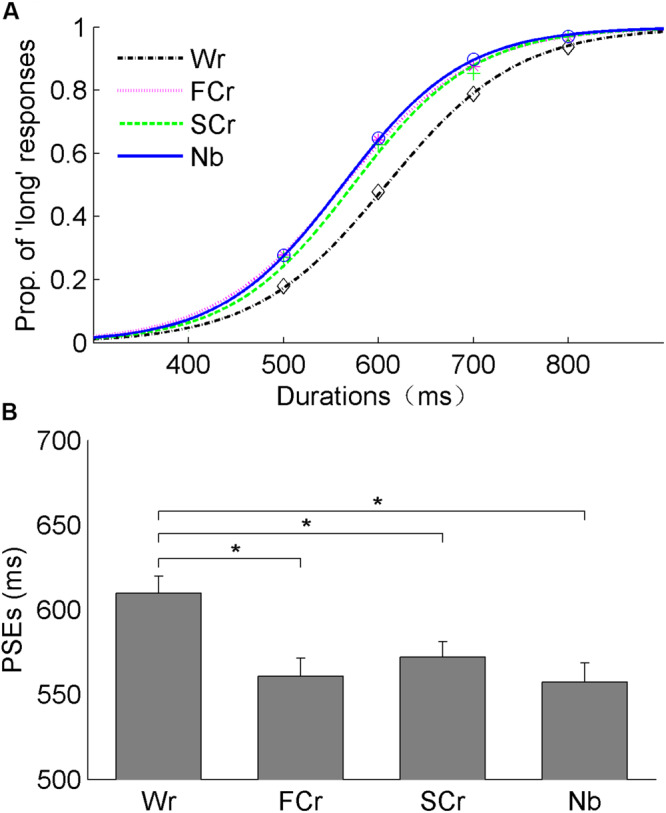
Results of Experiment 2a. **(A)** The fitted curves show the mean proportions of “long” responses against the comparison durations in the conditions of Wr, FCr, SCr, and Nb. **(B)** The bars show the mean points of subjective equality (PSEs) and their corresponding standard errors for the four experimental conditions (all *p < 0.05).

Experiment 2a compared the influence of word repetition with constituent character repetition on duration judgment. The mean PSEs (±SE) were 610 (±10), 561 (±11), 572 (±9), and 557 (±11) ms, for word repetition, first-character repetition, second-character repetition, and novel baseline conditions, respectively (Figure 3). A one-way repeated-measures ANOVA with Greenhouse–Geisser correction for PSEs revealed a significant main effect of repetition, *F*(1.936, 36.780) = 10.64, *p* < 0.001, η*_p_*^2^ = 0.36. Further pairwise comparisons with Holm–Bonferroni correction revealed that mean PSEs were significantly higher for the word repetition condition than the first-character repetition (mean differences: 49 ms, corrected *p* = 0.006), the second-character repetition (mean differences: 38 ms, corrected *p* = 0.024), and the novel baseline conditions (mean differences: 53 ms, corrected *p* = 0.006). The analysis failed to reveal any significant difference of PSEs between the first-character repetition and second-character repetition (corrected *p* = 0.212), between the second-character repetition and novel baseline (corrected *p* = 0.189), and between the first-character repetition and novel baseline (corrected *p* = 0.676). We applied the same statistical methods to analyze the JNDs across the experimental conditions; nonetheless, the results failed to show any statistically significant effect, Greenhouse–Geisser correction, *F*(1.957, 37.175) = 0.28, *p* = 0.84, η*_p_*^2^ = 0.015.

**FIGURE 4:**
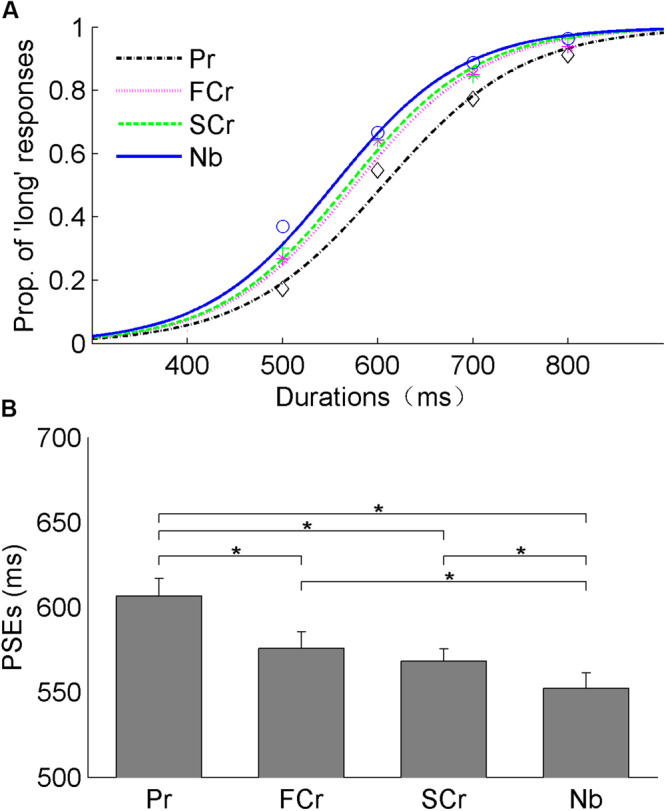
Results of Experiment 2b. **(A)** The fitted curves plot the mean judgments for pseudoword repetition (“Pr”), first-character repetition (“FCr”), second-character repetition (“SCr”), and novel baseline (“Nb”). **(B)** The bars indicate the mean points of subjective equality (PSEs), and their corresponding standard errors in each condition (all *p < 0.05).

As for Experiment 2b comparing the influence of pseudoword repetition with constituent character repetition on duration judgment, the mean PSEs (±SE) were 607 (±10), 576 (±9), 568 (±7), and 552 (±9) ms for pseudoword repetition, first-character repetition, second-character repetition, and novel baseline conditions, respectively (Figure 4). The ANOVA revealed a significant difference between the four experimental conditions, *F*(3, 54) = 16.16, *p* < 0.001, η*_p_*^2^ = 0.47. The *post-hoc* Holm–Bonferroni correction showed that mean PSEs were significantly higher for the pseudoword repetition condition as compared with the first-character repetition (mean differences: 31 ms, corrected *p* = 0.008), second-character repetition (mean differences: 39 ms, corrected *p* = 0.000), and novel baseline conditions (mean differences: 55 ms, corrected *p* = 0.000). Both PSEs for first-character repetition (mean differences: 24 ms, corrected *p* = 0.015) and for second-character repetition (mean differences: 16 ms, corrected *p* = 0.034) were significantly higher than the novel baseline. No significant difference in PSEs between the first- and second-character repetitions was found (mean differences: 8 ms, corrected *p* = 0.224). Last but not least, repetition conditions did not influence JNDs, *F*(3, 54) = 0.57, *p* = 0.64, η*_p_*^2^ = 0.03.

In short, our statistical analysis revealed a different pattern of results in Experiments 2a and 2b. That is, whereas in Experiment 2a (i.e., with word stimuli) neither first- nor second-character repetition conditions induced duration compression relative to novel baseline, the opposite was true for Experiment 2b (i.e., with pseudoword stimuli). More precisely, relative to novel baseline, pseudowords with both the first- and second-character repetition induced duration compression.

## Discussion

The current study investigated whether stimulus familiarity interacted with the repetition effect observed in a duration discrimination task using words and pseudowords. In Experiment 1, we observed that the repetition of Chinese words and pseudowords generated comparable duration compressions. In Experiment 2, we examined the constituent repetition effect when the standard–comparison stimuli shared the first or second constituent character. Interestingly, we did not find the constituent repetition effect for words (Experiment 2a). However, we did find a duration compression effect for constituent character repetition in pseudowords, although the strength of the compression was smaller than that of whole pseudoword repetition (Experiment 2b). We take these findings to imply that, (i) according to Experiment 1, familiarity alone does not impact the duration compression caused by the whole stimulus repetition, as words and pseudowords produced comparable duration compression. However, (ii) familiarity does mediate the effect of constituent character repetition in duration judgment as observed in Experiment 2.

The present study provides clear evidence in support of the repetition duration compression for both words and pseudowords, thus joining a stream of recent reports of the effect on duration judgment tasks (Birngruber et al., 2015; Cai et al., 2015; Matthews and Gheorghiu, 2016; Jia and Shi, 2017). Further, our study extends the current literature by demonstrating that duration compression is virtually comparable between words and pseudowords. Therefore, our findings indicate that a single repetition of an unfamiliar stimulus is sufficient to induce duration compression with a magnitude equivalent to that of a familiar stimulus. More specifically, the effect on duration estimation following the repetition of a stimulus is not proportionally influenced by the familiarity that the observer may have with it.

What is more, the new findings presented point to a different direction from those studies that employed lexical decision tasks, where greater repetition facilitation for words relative to pseudowords was observed (Henson et al., 2000; Fiebach et al., 2005; Orfanidou et al., 2006). The divergence between the current and previous studies is likely due to the different levels of processing specific to the requirements of each task. For instance, whereas lexical-related processing is essential in a lexical decision task, semantic processing seems not important in a duration judgment task. As an illustration, Jia and Shi (2017) showed in a recent study that orthographic and phonological information was more important for the repetition duration effect than semantic information. In their study, only phonological repetition between Chinese characters induced duration compression, whereas semantic repetition failed to induce the compression effect.

Concerning the effect of constituent character repetition in duration judgment when the standard–comparison pair shared a common character, we found disparate results for words and pseudowords. The perceived duration of the comparison pseudowords with an identical first or second character to that of the standard pseudoword was compressed relative to novel baseline, whereas the presentation of word pairs sharing constituent characters did not show this effect. The present findings suggest that familiarity modulates the effect of constituent repetition in the perception of time at least with Chinese words. This is contrary to studies that administered lexical decision or identification tasks, wherein the effect of constituent repetition for word was shown when the standard and comparison stimuli shared constituent characters (Zhou et al., 1999; Tsang et al., 2014; Wu et al., 2016). A possible explanation for this disparity stems from the idea that semantic-related judgment of words, such as lexical decision, requires a deeper level of processing because each constituent morpheme must be interpreted. In a duration judgment task, however, lexical interpretation is “overwritten” by time estimation as the focus of the task is on how long a stimulus is present on the screen rather than on its semantic processing. Over and above that, words are composed of constituent characters with a familiar linkage, leading to a more effective performance when words are processed holistically, as compared with when they are processed at the morpheme level. On this note, it is possible that when the standard and comparison words share only a constituent character in a duration judgment task, the latter is processed as a novel stimulus. Conversely, as the structure of pseudowords is formed by unrelated characters, it is likely that each stimulus is processed at the character level. This idea is supported by the findings that extensive prior exposure to a stimulus enhanced the associations among its features compared with exposure to an untrained stimulus (Meyer and Olson, 2011; Grotheer and Kovács, 2014). In other words, familiar stimuli (e.g., words) tend to be processed at an integrated level, whereas unfamiliar stimuli (e.g., pseudowords) are processed at the component level.

In order to explain the repetition duration effect, the attentional account assumes that unexpected stimuli capture more attention than do expected stimuli, thus resulting in longer subjective duration (Tse et al., 2004; Matthews and Gheorghiu, 2016; Matthews and Meck, 2016). In this specific regard, the attentional account supports our results as we found that the duration of novel words/pseudowords were judged as being longer than that of repeated stimuli (Tse et al., 2004). However, the attentional account does not explain the fact that the influence of constituent repetition led to different outcomes in the word and pseudoword conditions. Moreover, as per the attentional-gate model, when more attentional resources are given to the non-temporal features of an unexpected stimulus, less attention is devoted to the temporal features. This process may skip the temporal pulses otherwise accounted for, thus shortening the subjective duration of the unexpected stimulus (Zakay and Block, 1997; Ulrich and Bausenhart, 2019). This outcome is the opposite effect commonly observed in the classic duration compression of repeated stimuli. Similarly, the arousal account also fails to offer a satisfactory explanation for the divergent findings regarding constituent repetition of words and pseudowords. In other words, JNDs indicating the sensitivity to temporal discrimination did not show significant changes among different experimental conditions. Therefore, the current results cannot be thoroughly explained by either attentional or arousal accounts.

A further, possible explanation for the current results concerns the influence of memory and decision under the framework of the internal clock (Gibbon, 1991; Wearden, 1992). For instance, memory-related factors such as the ISI between the standard and comparison stimuli have been shown to affect the repetition effect, as demonstrated in a study by Matthews (2015) where the repetition effect in the short (e.g., 306 ms) and long (e.g., 2,000 ms) ISI conditions were compared. The results showed that the repetition effect only occurred in the short-ISI condition, indicating that the effect was short-lived. Consequently, it is unlikely that memory-related factors influenced the present findings, as short ISIs (300–500 ms) were used across all experimental conditions. In the same vein, it is improbable that the present results reflect a decision bias. This is because the repetition duration effect has been manifested in other judgment tasks such as the equality judgment task, which is not affected by response bias (Birngruber et al., 2014). Over and above that, there is a consensus that the repetition duration effect is rather a response to perceptual bias (Birngruber et al., 2014; Matthews and Gheorghiu, 2016; Ulrich and Bausenhart, 2019).

An alternative to the internal clock mechanism, namely, the repetition suppression account, argues that neural responses to repeated words/pseudowords get suppressed, which then leads to a reduced perceived duration (Grill-Spector et al., 2006; Noguchi and Kakigi, 2006; Eagleman and Pariyadath, 2009) where the size of the repetition duration compression is relative to the difference between the comparison and standard stimuli. Although this view might explain the differential magnitudes of duration compression between whole-repeated and constituent-repeated pseudowords, it does not explain the lack of duration compression for constituent repeated words. Indeed, some neuroimaging studies have shown that neural responses are not only suppressed but also enhanced for repetition (Segaert et al., 2013), indicating that the neural responses do not always reflect the perceived duration.

Last but not least, the predictive coding account suggests that the expected stimulus produces a shortening of subjective duration to optimize coding efficiency, a process within which the magnitude of the repetition duration effect depends on the similarity between the standard and comparison stimuli (Rao and Ballard, 1999; Friston, 2005). Specifically, the fewer the prediction errors (from the difference between the predicted and actual signals),the shorter the subjective duration perceived. Applied to the present study, we observed a discrepancy between the standard stimulus and two comparison conditions, namely, the constituent repeated and novel stimulus. Thus, prediction errors differed among three experimental conditions: no prediction error for whole-repeated stimuli, partial prediction error for the constituent repeated stimuli, and the most prediction errors for novel stimuli. In other words, the subjective duration of whole-repeated stimuli was perceived as being shorter than the other two stimuli, whereas the presentation of the constituent repeated stimulus was perceived as shorter than the novel, in the pseudoword condition. However, the constituent repetition of words did not show a duration compression. We suggest that the implicit expectation may encompass words at a configural and/or holistic level in duration judgment. As a result, word pairs sharing one constituent character evoke prediction errors similar to that evoked during the judgment of novel word pairs. On the contrary, when participants see the standard pseudoword with no relation between constituent characters, naturally, they expect an unfamiliar comparison stimulus with similar characteristics. However, when standard and comparison pseudowords share a constituent character, the surprise prediction error is only partial. Consequently, the compression effect for pseudowords is reduced compared with whole words. Consequently, this process places the predictive coding account as a more comprehensive explanation for the present repetition duration compression than the aforementioned accounts of attentional/arousal and repetition suppression (Jia and Shi, 2017).

In summary, the present study provides clear evidence that the repetition of words and pseudowords promotes analogous duration compression, indicating that familiarity *per se* does not interact with the effect of the whole repetition in duration judgment. Moreover, we revealed that when the standard and comparison stimuli shared a constituent character at the first or second position, there was a repetition compression for pseudowords; however, it was reduced in relation to the whole pseudoword repetition. By contrast, the constituent repetition of words did not show the compression effect, as words with only a constituent repetition might have been processed as a novel word altogether. We suggest this outcome indicates that pseudowords are processed at the component level whereas words are processed holistically in a duration judgment task. Therefore, we claim that familiarity interacts with the constituent repetition effect, but differently for words and pseudowords. Thus, the pattern of results across the two experiments favors the idea that the magnitude of the repetition compression depends on the difference between the actual signal and prediction.

## Data Availability Statement

The datasets generated for this study are available on request to the corresponding authors.

## Ethics Statement

The studies involving human participants were reviewed and approved by the Ethics Committee of Jiangnan University. The patients/participants provided their written informed consent to participate in this study. Written informed consent was obtained from the individual(s) for the publication of any potentially identifiable images or data included in this article.

## Author Contributions

LJ, XZ, and XW developed the design of the study. CD collected the data. LJ, XZ, and LW performed the data analyses. LJ, XZ, LW, and XW contributed to the writing and drafting the manuscript.

## Conflict of Interest

The authors declare that the research was conducted in the absence of any commercial or financial relationships that could be construed as a potential conflict of interest.
